# Influence of gut microecology in the development of malignant tumors and its potential therapeutic application: A review

**DOI:** 10.1097/MD.0000000000034274

**Published:** 2023-07-07

**Authors:** Jin-Ping Qian, Bing Jiang, Xu-Dong Lei, Le-Le Tian, Ying Zhou, Jing-Quan Teng, Jia Yue, Jin-Juan Li, Yan Zhang

**Affiliations:** a Gansu Key Laboratory of Traditional Chinese Medicine Excavation and Innovative Transformation, Gansu University of Traditional Chinese Medicine, Lanzhou, Gansu, China; b Department of Integrated Chinese and Western Medicine, Gansu University of Traditional Chinese Medicine, Lanzhou, Gansu, China; c Gansu Provincial Cancer Hospital, Lanzhou, Gansu, China; d Northwest Collaborative Innovation Center for Traditional Chinese Medicine Co-constructed by Gansu Province & MOE of PRC, Lanzhou, Gansu, China.

**Keywords:** gut microbiota, malignant neoplasm, mechanism of carcinogenesis, metabolites, therapeutic strategy

## Abstract

A microbial ecosystem is a complex community of multiple bacterial interactions. The potential role of gut microbiota in human health has already attracted the attention of many researchers. Dysregulation of the gut microbial community has been suggested to be closely associated with the progression of various chronic diseases. Malignant neoplasms represent a major global health burden and are now the leading cause of death. The formation of tumors is often thought to be influenced by genetic and environmental factors. Recent research advances have indicated that multiple malignancies may also be attributed to the gut microbiota. In this review, we highlight the complex interactions between gut microbes and their metabolites, as well as the potential impact of gut microecology on the occurrence and development of tumors. In addition, potential strategies for targeted therapy of tumors using gut microecology are discussed. In the near future, intestinal microecology is likely to be used for early screening of tumors and subsequent clinical treatment.

## 1. Introduction

Malignant tumors are a major global health burden and have now become the first cause of death.^[1]^ The formation of tumor formation is often thought to be influenced by genetic and environmental factors. Moreover, following the improvement of human living standards, a variety of factors, such as changes in dietary habits, endocrine disorders, and immune dysfunction, may also increase the incidence and mortality of cancer. And with the deepening of antitumor research, treatment methods are also gradually developing. Following traditional surgery, radiotherapy, and chemotherapy, new treatments (consisting of interventional therapy, molecular targeted therapy, cellular therapy, and immunotherapy) have been widely used in the treatment of cancer.^[2–4]^ However, the prognosis of most patients remains very poor, which is associated with some factors, such as poor response to treatment regimens, more toxic side effects, and multi-drug resistance.^[5,6]^ Therefore, exploring effective and safe treatment strategies still remains a pressing clinical problem.

Recent studies suggest that a potential link is existed into the relationship between gut microbiota and cancer.^[7–9]^ In fact, the gut microecological constantly accompanies our life. From birth, microorganisms settle in the intestines of our human body, and a large number of microorganisms are present in the food and air, which we are exposed to daily, and even in many parts of our skin, mouth, and intestines. Thus, the human body is a super organism containing microorganisms. In addition, the human intestine also provides a good habitat for microorganisms. According to statistics, the number of microorganisms in the adult intestine can be as high as 1014, close to 10 times the number of human somatic cells. The mass can reach 1.2 kg, close to the mass of human liver. The number of genes is about 100 times that of the human body itself, so as to have the metabolic functions that the human body does not have. In other words, the gut microecology can be viewed as an interacting complex biome, or even collectively viewed as the largest endocrine organs. Although the gut microbiota provides many necessary and beneficial physiological processes for the human body, such as the digestion and absorption of nutrients and the synthesis of some vitamins, a large amount of evidence shows that the dysregulation of gut microbiota can play a regulatory role in the development of adverse phenotypes. In particular, distinct changes in the structure and function of microbial communities are associated with a variety of disease states, such as cardiovascular diseases, gastrointestinal diseases, gynecological diseases, malignant tumors, and so on.^[10]^

This review elaborates on the association between gut microecology and tumors from the following aspects: Firstly, the main components of intestinal microecology (intestinal microbiota and metabolites) are described. Secondly, the role of intestinal microecology inoccurrence and development of tumors is discussed. Finally, the potential therapeutic strategies that affect tumor progression by regulating intestinal microecology are discussed. Here, our purpose is to study the impact of changes in the intestinal microecology on tumor progression and to provide new insights for early screening and subsequent treatment of cancer.

## 2. Gut microbial flora

There are many kinds of microorganisms in the human gastrointestinal tract, which are called intestinal flora. Intestinal flora is combined in a certain proportion, and each bacterium restricts each other and depends on each other, so as to form an ecological balance in quality and quantity. The gut microbiota can respond to the nutrients consumed by the host through various hormones and also to the state of the host, resulting in compounds used to signal the system in the human body, including neurotransmitters (such as γ-aminobutyric acid), amino acids (such as tyrosine and tryptophan, in which tryptophan can be converted into molecules of controlling mood, dopamine, and serotonin) and many other substances. According to their different physiological functions, gut microbial flora can be broadly divided into 2 categories: the native flora, also known as the normal flora, is composed of fairly fixed bacteria that regularly settle in some specific parts of the body and become an integral part of the body. In the long-term evolutionary process, through individual adaptation and natural selection, different species in the normal flora, between the normal flora and host, and between the normal flora, host, and environment are always in a state of dynamic balance state, and then form an inter-dependent and mutually restricted system. Therefore, under the normal condition of the human body, the performance of normal flora on the host is often nonpathogenic, but has important physiological functions, such as immune regulation, detoxification, avoiding the invasion of pathogens, providing nutrients (vitamins, amino acids, lipids, and carbohydrates), anti-aging and antitumor. The foreign flora, also known as the transit flora, is composed of nonpathogenic or potentially pathogenic bacteria, mainly from the surrounding environment or other habitats of the host, which can remain in the host for hours, days, or weeks. When the normal flora of the human body is disturbed, the transit flora can multiply in a short period of time, so as to cause disease.^[11]^

Different segments of the intestine have different dominant flora, and there is specific communication for chemical signals between flora (such as food chain, informational transmission, gas transmitters, and so on) and between host and flora in each part of the intestine. These “signaling molecules” include low-molecular-weight metabolites, peptides, proteins, and so on. They can even replace some indirect immunoregulatory pathways, which also provide a feasible basis for the treatment of diseases through immunoregulatory pathways.^[12]^ In addition, abundant bacteria in the gut contribute to normal digestive function, and 98% of these gut microbes can be divided into 4 phyla, including firmicutes, bacteroidetes, proteobacteria, and actinobacteria. Numerous studies have shown that some gut microbes (such as Escherichia coli, bifidobacterium, eubacterium, lactobacillus, bacteroides, and so on) are involved in the biological transport of natural products,^[13]^ which seems to provide a theoretical basis for exerting significant pharmacological effects by regulating gut microecology.

### 2.1. E. coli

*E. coli* is a gram-negative genus, nonsporulating, facultative anaerobic bacterium that is mainly distributed in the gut of vertebrates.^[14]^ Some *E. coli* can produce glycosidases involved in the transformation of exogenous substances, thereby producing their beneficial effects. For example, Han DH et al^[15]^ have found that *E. coli* can produce β-glucuronidase to hydrolyze the glycosidic bond in baicalin, so as to produce baicalein. At the same dose, baicalein inhibits histamine-induced scratching behavior more effectively than baicalein and has anti-oxidant and anti-inflammatory effects by inhibiting Nrf2/ARE/HO-1 as well as NF-κB signaling pathway activity.^[16]^ Some *E. coli* strains also have high curcumin transforming activity. Studies have shown that *E. coli* can promote the high expression of NADPH-dependent curcumin-dihydrocurcumin reductase, thereby reducing curcumin to dihydrocurcumin and tetrahydrocurcumin. Subsequently, dihydrocurcumin and tetrahydrocurcumin reduce triglyceride levels in cells by regulating mRNA and protein expression levels of SREBP-1C and PPARα, and inhibit overproduction of fat in the liver in a dependent manner, so as to bring therapeutic benefits in fatty liver degeneration.^[17]^ Another report has shown that *E. coli* can activate the activity of cinnamoyl esterase and release hydroxycinnamic acid by hydrolyzing conjugated hydroxycinnamic acid and free hydroxycinnamic acid esters, so as to show properties of anti-oxidant and anticancer.^[18]^ Therefore, a good understanding of the genetic and biochemical characteristics of *E. coli* may facilitate the synthesis of natural product derivatives with various health activities in vitro.

### 2.2. Bifidobacteria

Bifidobacteria are widespread and abundant genera within the phylum of actinobacteria and are one of the first colonizers of human gut microbes.^[19]^ Certain species of bifidobacteria can produce phenolic acids by expressing feruloyl esterases. For example, Pang C et al^[20]^ have found that feruloesterase from bifidobacterium animalis hydrolyzes chlorogenic acid to caffeic acid, which prevents acetaminophen-induced acute liver injury in mice by increasing Nrf2 transcription. Some bifidobacteria can also hydrolyze 6 major bile salts in the host, so as to regulate the metabolism of bile acid and reduce cholesterol levels in the body.^[21]^ In addition, β-glucosidase produced by bifidobacteria can cleave glycosides at C-3 and C-20 positions of ginsenoside Rd to generate deglycosylated ginsenosides. At the same dose, deglycosylated ginsenoside is more effective than ginsenoside Rd in inhibiting the growth of tumor cells, inducing tumor cell death as well as abnormal differentiation of retrograde tumor cells.^[22]^ Thus, bifidobacteria may help stimulate the potential benefits of these natural products, so as to better exert pharmacological effects.

### 2.3. Eubacterium

Eubacterium is not only a gram-positive bacterium, but also is one of the core genera of human gut microbiota, and has shown extensive colonization of the human gut.^[23]^ Some eubacterium can produce glycosidases, reductases, and so on, and are involved in the metabolism of exogenous substances. For example, chalcone isomerase from eubacterium and flavanone flavanol cleavage reductase produce chalcones and dihydrochalcones by degrading certain flavonoids, while dihydrochalcone and its metabolites have anti-inflammatory and antioxidant effects, they can reduce the secretion of pro-inflammatory cytokines and rescue lipopolysaccharide (LPS)-induced oxidative phosphorylation.^[24]^ Kim DH et al^[25]^ have found that β-glucuronidase in eubacteria hydrolyzes glycyrrhizic acid to 18β-glycyrrhetinic acid. However, 18β-glycyrrhetinic acid prevents airway allergic inflammation by inhibiting NF-κB phosphorylation and enhancing Nrf2/HO-1 signaling pathway.^[26]^ Hence, these metabolic transformations suggest potential benefits of eubacterium for treating diseases.

### 2.4. Lactobacillus

Lactobacillus belongs to the phylum of firmicutes, balances microbial communities, and protects the gastrointestinal mucosa.^[27]^ Some lactobacillus species are rich in metabolic enzymes, such as α-rhamnosinase, tannase, gallate decarboxylase, and so on, which can transform into exogenous substances. For example, lactobacillus hydrolyze gallates by expressing tannase, protocatechuates with short aliphatic alcohol substituents, and complex gallotannins to produce gallic acid.^[28]^ Whereas gallic acid plays a protective role in LPS-induced inflammation and oxidative stress by inhibiting MAPK/NF-κB pathway and activating Akt/AMPK/Nrf2 pathway.^[29]^ In addition, Fang C et al^[30]^ have observed that gallic acid and pyrogallol are produced through the degradation of gallosides by galloylglycine metabolizing enzymes in lactobacilli, which implies the potential role of interaction between prebiotics and probiotics in preventing diet-induced metabolic disorders. Another report has shown that lactobacillus with daidzein reductase activity can reduce daidzein to dihydrodaidzein,^[31]^ while dihydrodaidzein ameliorates osteoporosis by inhibiting NF-κB activation and MAPK phosphorylation.^[32]^ Therefore, these findings open up new perspectives on the role of lactobacillus in pharmaceutical and food applications to promote health.

### 2.5. Bacteroides

Members of the bacteroides genus are gram-negative obligate anaerobes, accounting for 25% of the total number of bacteria in the colon, and playing multiple roles in the human intestinal bacteria group.^[33]^ Bacteroides has a series of hydrolases and participates in the association with its microbial neighbors through the transformation of foreign substances. In vitro co-culture experiments have shown that some bacteroides species participate in the bio-transformation of flavonoids. For example, bacteroides can express α-l-rhamnosidase and β-Rutinosidase, which are used to hydrolyze rutin into quercetin 3-o-glucoside, quercetin, and leucanthin.^[34]^ Among them, quercetin 3-o-glucoside is better absorbed than other forms of quercetin and can inhibit NF-κB and MAPK signaling pathways to inhibit inflammation in colitis mice.^[35]^

**Figure 1.**
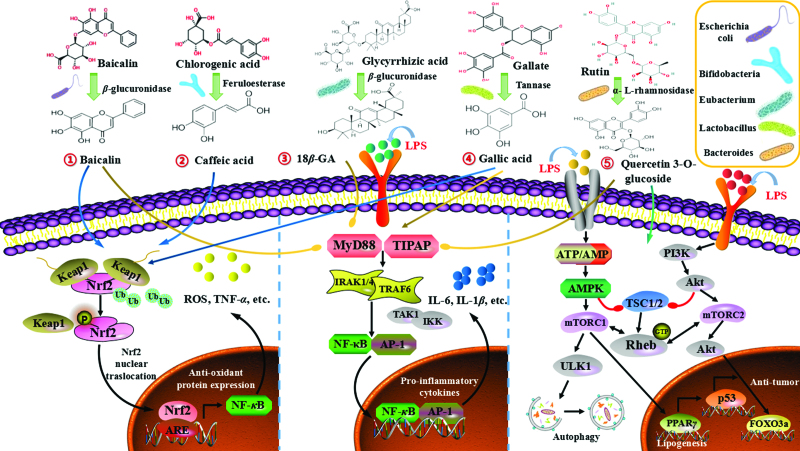
Potential application of key enzymes produced by intestinal microorganisms in bio-transformation of natural products.

These evidence show that natural products are bio-transformed under the action of enzymes produced by different gastrointestinal microorganisms, and produce natural derivatives with different functional activities, so as to better play a pharmacological role in related diseases (Fig. 1). Therefore, it is particularly important to understand the whole process of natural products in human body to assess their impact on human health.

## 3. Gut microecological metabolite

**Figure 2.**
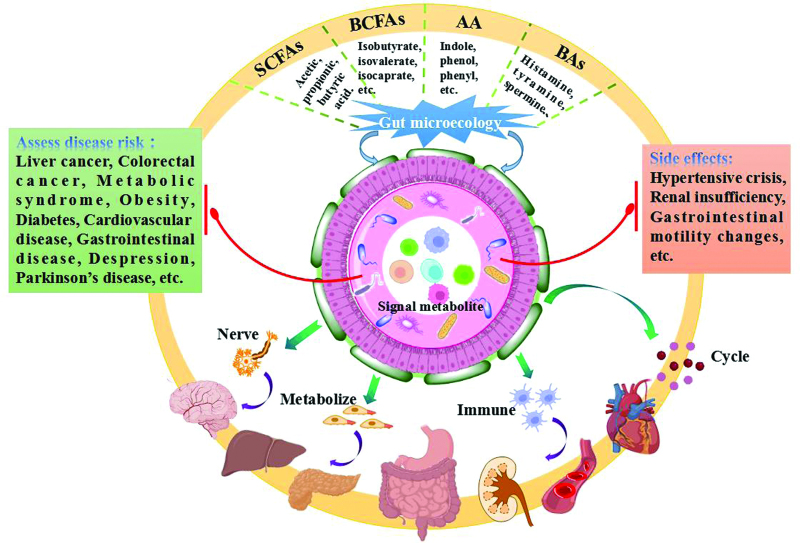
Central hubs for gut microbial metabolites to coordinate the regulation of multiple factors.

The gut microbiota generates a very diverse repertoire of metabolites, which from fermentations of exogenous undigested dietary components reaching the colon to endogenous compounds produced between microbes and hosts. Moreover, the monolayer epithelial cells that form the interface between the host and microbial mucosa enable microbial metabolites to better enter host cells, and interact with the host cells, so as to affect the immune response and disease risk (Fig. 2).

### 3.1. Short-chain fatty acids (SCFAs)

Although the contribution of SCFAs to human circulatory metabolism is limited, they are the most important way in the interaction between host and microorganism metabolism. It is well-known that nutrients for the growth of human colon microbiota, including endogenous mucins, glycoproteins, proteins, oligopeptides, oligosaccharides, and dietary polysaccharides, which evade digestion by the host and are then utilized by microorganisms, while the main products of microbial fermentation in the human colon are acetic acid, propionic acid, and butyric acid. A large number of data have shown that their ratio in the intestine is 60: 20: 20 (acetic acid: propionic acid: butyric acid). Accumulated concentrations of these compounds in the lumen distal to the gut can exceed 100 mM, and nearly 95% of the resulting SCFAs are absorbed by the host, while contributing almost 6 to 10% to energy requirements in humans. Acetic acid (or acetic acid) and propionic acid are rapidly absorbed in the colon and delivered to the liver via the hepatic portal vein for metabolism at the distal end of the intestine.^[36]^ Isotope labeling suggests that acetate is rapidly metabolized, contributes to gluconeogenesis, and enters the tricarboxylic acid cycle, so as to promote lipogenesis.^[37]^ Acetate can be metabolized in systemic tissues, while propionate is only extensively metabolized in the liver and acts as a gluconeogenic substrate in the liver. Isotope labeling in mice suggests a high degree of propionate conversion by the liver, and 62% of propionate is converted to glucose in the intestine.^[38]^ The data on sudden death patients shows that the utilization rate of propionate is 30%.^[39]^ However, despite the high utilization rate of propionate, its contribution may be little to glucose requirements in humans. In contrast, butyric acid is the preferred substrate for proximal metabolism. Butyric acid is preferentially absorbed and undergoes β-oxidation through colonocytes, which is a process of consuming oxygen and activating colonocytes. Studies on isolated rat enterocytes have shown that 70 to 80% of oxygen utilization is due to β- oxidation of butyric acid, while the utilization of butyric acid by cells is higher than that of other metabolic substrates.^[40]^ The importance of butyrate metabolism for colon-forming cells has also been demonstrated that germ-free mice can exhibit a situation of impaired metabolism and increase autophagy rates in the absence of butyrate for prolonged periods, and can be rescued by the bacteria of increasing butyric acid or producing butyric acid.^[41]^ Hence, it is particularly important to understand the metabolic process of SCFAs and their fermented products in human body to assess their impact on liver, colorectal-related tumors, and metabolic damage.

### 3.2. Branched-chain fatty acids (BCFAs)

BCFAs are produced by the degradation of valine, leucine, and isoleucine. The end products of these pathways include isobutyrate, 2-methylbutyrate, isovalerate, and isocaprate. Although the abundance expression of these compounds in feces is lower than SCFAs, they can still affect the physiology of human intestine. Recent studies have shown that these compounds have local effects on gastrointestinal-related cells and affect the physiology of the host.^[42]^ For example, isovaleric acid is specifically recognized by chemosensory receptors on intestinal chromaffin cells, so as to activate neurons, indicating that the metabolites produced in the intestine can be perceived by the intestinal nervous system and change gastrointestinal motility and other processes.^[43]^ Evidence suggests that BCFAs can accumulate in the host serum. Under the action of isovaleric acid, circulating levels are close to 40 μM. It can be argued that this concentration is exclusively produced by microorganisms because isovaleric acid intermediates in human leucine metabolism are considered to be sequestered in mitochondria as isopentyl coenzyme A.^[44]^ Other studies have shown that microorganisms-derived BCFAs, especially isovaleric acid, play an important role in cholesterol synthesis, and they can regulate mitochondrial β-oxidation of pyruvate, so as to alter the adipogenesis of adipocytes, suggesting that the metabolism of branched-chain amino acids in the host may be related to obesity, metabolic syndrome, and diabetes.^[45]^

### 3.3. Aromatic acids

Many studies have identified aromatic compounds in the gut that are produced by microbiota metabolism.^[46]^ Aromatic compounds are produced by the microbiota, containing indole, phenol, and phenyl groups, which can participate in the metabolic processes of human body, including sulfation, glucuronidation, and amino acid conjugation. The sources of aromatic metabolites produced by microorganisms mainly include non-digested proteins, mucus, aromatic amino acids in intestinal secretions, and exfoliated epithelial cells, as well as dietary polyphenols and plant secondary metabolites from fruits, vegetables, coffee, tea, and wine. Among them, the metabolism of aromatic amino acids can produce toxins that lead to uremia, such as indole sulfate, p-cresol sulfate, and phenylacetylglutamine. Some data have shown that the metabolites of aromatic amino acids can also contribute to the improvement of cardiovascular disease but cause renal dysfunction.^[47]^ In addition, indolepropionic acid is a tryptophan metabolite produced by a few intestinal bacteria, which has an important impact on the intestinal barrier function of host.^[48]^ Other tryptophan metabolites, collectively known as indole, seem to play an important role in regulating tissue repair after injury and immune homeostasis in the intestine.^[49,50]^ It can be seen that by regulating the whole process of aromatic compounds and their metabolites in the human body, it has potential research value for the treatment of cardiovascular diseases, kidney diseases, and gastrointestinal diseases.

### 3.4. Biogenic amines (BAs)

The sources of BAs in humans mainly cover endogenous, dietary, and microbial sources, while they mediate a variety of biological activities. For example, histamine, an endogenous BA, is an effective vasodilator released by degranulation mast cells activated by allergens that bind immunoglobulin E receptor and can be used clinically for cardiovascular disease.^[51]^ Tyramine, a BA found in cheese, is usually metabolized by monoamine oxidase. Ingestion of large amounts of tyramine has been found to cause a life-threatening hypertensive crisis in patients who take MAO inhibitors for depression or Parkinson disease.^[52]^ Spermine, a BA of dietary, endogenous, and microbial origin, is closely associated with regulation of immune homeostasis and maintenance of healthy microbiota.^[53]^ Overall, it is currently only recognized that BAs are able to be produced by gut microbes, but the understanding of their human health effects is not yet fully known.

## 4. Role of intestinal microecology in the process of carcinogenesis

**Figure 3.**
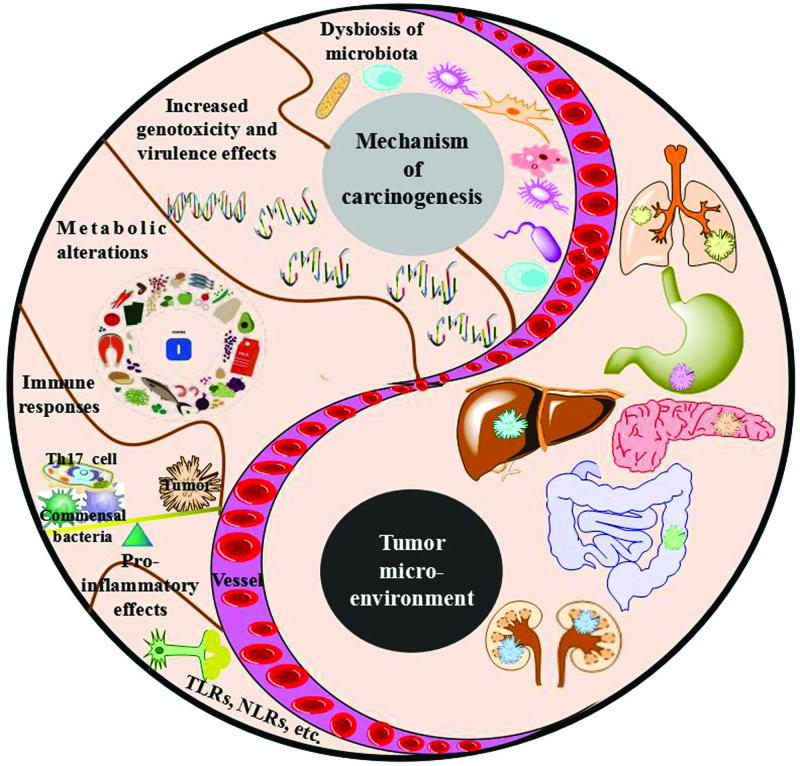
Dysbiosis in gut regulates susceptibility to the tumor microenvironment at multiple levels.

The symbiotic relationship between host and microbiota is based on multi-level barriers and immune sensing systems. Once barrier defects or immunodeficiency occur, microbial components and bacterial translocation are perturbed, leading to interactions between the microbiome and epithelial cells or the immune system, which may promote dysregulation of the microbiota and subsequent chain reactions, so as to cause carcinogenesis. In addition, inflammatory signaling activation, changes in dietary habits, infection, NOD2 deficiency, and other factors may also lead to microbiota dysregulation, so as to cause a decrease in co-microorganisms and an increase in inflammation-induced bacteria, which in turn leads to multiple levels of carcinogenesis. More and more studies have shown that microflora dysregulation regulates susceptibility to malignancy at multiple levels, including dysbiosis of microbiota, increased genotoxicity, increased virulence effects, metabolic alterations, immune responses, and pro-inflammatory effects^[54]^ (Fig. 3).

### 4.1. Dysbiosis of microbiota

Common tumor-associated bacteria are usually fusobacterium, *E. coli*, bacterium fragilis, porphyromonas, and proteus. Among them, excessive fusobacteria are often thought to activate carcinogens, so as to promote the formation of tumor. Actinobacteria (bifidobacteria) and firmicutes (enterococcus) in the gut are the largest biomarkers of tumors and their abundance expression levels are also significantly different in cancer patients. Zheng Y et al^[55]^ have analyzed the intestinal flora of 42 patients with early lung cancer and 65 healthy people, it is confirmed that the abundance of bacillus in the intestine of patients with early lung cancer is increased, while bifidobacterium and fecal bacilli are more abundant in the control group. Lee SH et al^[56]^ have found that Veillonella and Megasphaera can be used as potential biomarkers in early diagnosis of lung cancer through characteristic analysis of the microbiome in patients with lung cancer and benign lung masses.

### 4.2. Increased genotoxicity and virulence effects

Regarding genotoxicity, Wang XM et al^[57]^ found that reactive oxygen species (ROS) could mediate the responses of double-stranded DNA damage. And recent studies have shown that dysregulation of the microbial flora alters the levels of ROS, which causes double-stranded DNA damage response and carcinogenesis. Bacterial toxins, such as cell swelling toxin, cytotoxic necrosis factor 1, and fragilis toxin, are identified as mediators causing double-stranded DNA damage responses.^[58]^ In addition, bacteria-driven hydrogen sulfide and superoxide radicals have been found to be responsible for genomic instability.^[59]^ Another study has reported that fusobacterium adhesin A secreted by fusobacterium can interact with the E-cadherin gene, so as to promote the metastasis of tumor by regulating Catenin signaling pathway.^[60]^ Regarding virulence effects, Burns MB et al^[61]^ have found that virulence-associated with bacterial genes occur enrichment in the tumor microenvironment, which may be dependent on the genomes of Clostridium and Providencia.

### 4.3. Metabolic alterations

The microbiota has been proven to participate in the regulation of metabolic processes in the host, which is associated with detoxification, hormone production, bile acid secretion, nutritional status, and vitamin levels. Previous studies have shown that the bacterial microbiota contributes to producing acetaldehyde, which is an important carcinogen.^[62]^ Moreover, deoxycholic acid, an obesity-induced gut microbial metabolite, contributes to the impact of the development of obesity-associated hepatocellular carcinoma.^[63]^ And recent studies have found that dietary fiber can promote the fermentation of gut microbiota into SCFAs, which have anti-inflammatory effects and can reduce the incidence of colon cancer and breast cancer.^[64]^

### 4.4. Immune responses

The microbiota plays an important role in the formation of adaptive immunity throughout the life cycle. Therefore, the central role of how microbiota regulates the immune response of tumors must be taken seriously. Enterotoxigenic bacteroides fragilis activates transcriptional activator 3 through a selective T-helper 17 (Th17) response, suggesting that human commensal bacteria can induce cancer through a Th17-dependent pathway.^[65]^ In addition, the expression of calcineurin and nuclear factor of T cytokines is upregulated by activating tumor-associated with microbial flora and Toll-like receptor (TLR) signaling, so as to maintain the survival and proliferation of cancer stem cells.^[66]^ Another study has shown that microbially-derived butyrate can expand regulatory function of T cells by activating forkhead box P3 and G protein-coupled receptors.^[67]^ In addition, pathological microbiota can promote the expression of IL-17C in epithelial cells of patients with chronic obstructive pulmonary disease (COPD), and induce the growth of neutrophils in tumor microenvironment, so as to induce inflammation and cause multi-level carcinogenesis.^[68]^ Take the occurrence and development of lung cancer. At present, the study on the symbiotic mechanism between lung microbiology group and lung cancer is still in the preliminary stage. Chronic pulmonary inflammation, such as COPD, is defined as a risk factor for lung cancer.^[69]^ Recent studies have revealed the role of lung microbiotic variation in lung cancer development. Weeks JR et al^[70]^ have found that the epithelial cytokine IL-17C induced by nontypeable Haemophilus influenzae in COPD patients can mediate the proliferation of tumor via increasing neutrophil inflammation. Another study has shown that smoking exposure combined with nontypeable *H. influenzae* can promote the metastasis and proliferation of lung cancer because smoking can promote bacterial translocation.^[71]^ Moreover, IL-6 may play an important role in lung cancer by promoting COPD-like inflammation.^[72]^ Microorganism-induced Th17 cells can promote the proliferation and angiogenesis of lung cancer cells.^[73]^ In general, these evidences may be the key to inhibiting tumor development by maintaining homeostasis of host immune.

### 4.5. Pro-inflammatory effects

Activation of inflammatory pathways, such as microbiota-associated molecular patterns or PRR signaling, not only senses the status of microbiota but also promotes the occurrence and development of tumors. More and more evidence have shown that the activation of TLRs can play a key role in the progression of colon cancer, gastric cancer, liver cancer, and pancreatic cancer.^[74]^ Among them, knockout of TLR4 gene can inhibit carcinogenesis in mice, and the mechanism may be related to promoting the death of tumor cells via inhibiting NF-κB pathway, signal transducer, and transcriptional activator 3. Interestingly, TLR modulation associated with cancer also increases susceptibility to specific infections, so as to promote oncogenic processes by increasing the expression of certain TLRs. In addition, the microflora induces MYD88 in myeloid cells, which triggers IL-23 signaling, accelerates the progression of tumor, and promotes the development of tumor IL-17 responses.^[75]^ And a recent study has shown that the lack of IL-17C can promote the growth and metastasis of lung cancer models.^[76]^ Moreover, certain potential factors also play an important regulatory role in the process of inflammatory infection leading to tumors. For example, Nod-like receptors (NLRs) are a PRRs subfamily located on the cell membrane, which can activate a series of defensive mechanisms to fight against invasive bacteria.^[77]^ The protective effect of NOD1 is mainly through the barrier preventing the transition from inflammation to carcinogenesis.^[78]^ NOD2 also plays an important role in the regulation of microbial flora and reduces susceptibility to colorectal cancer. When the NOD2 gene is knocked out in mice, it can lead to bacterial overload and inflammatory responses.^[79]^ In addition, deficiency of NLRP6 in mice decreases IL-18 production, so as to increase susceptibility to colorectal cancer,^[80]^ and similar results are also found in NLRP12-deficient mice,^[81]^ implying NLRP6 and NLRP12 play an important role in dysregulation of microbiota and carcinogenesis.

## 5. Gut microecological targeted therapy for tumors

**Figure 4.**
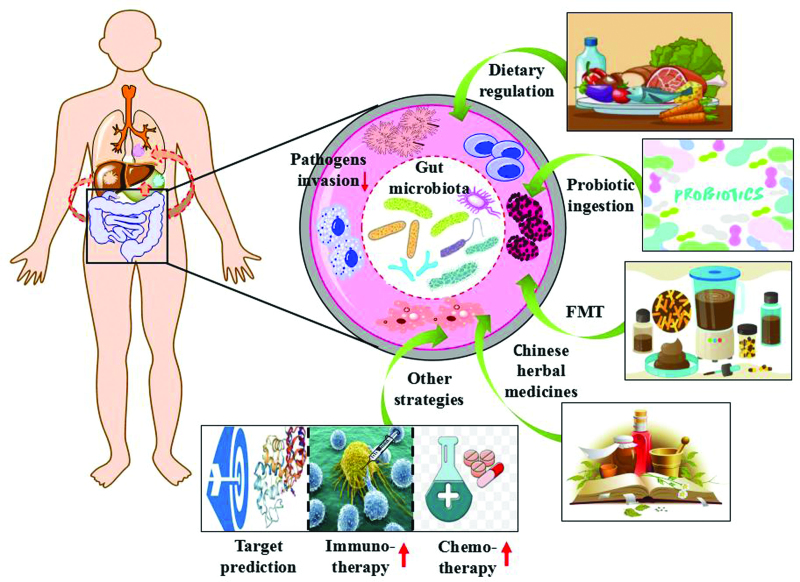
Improving potential therapeutic strategies associated with malignant tumors through gut microecology.

Increasing evidence suggests that intestinal microecology plays an important role in the progression of malignant tumors.^[82]^ Therefore, the regulation of intestinal microecology has become an ideal target for the prevention and treatment of diseases. At present, some therapeutic strategies, such as dietary regulation, probiotic ingestion, fecal microbial transplantation (FMT), and the application of Chinese herbal medicines, can re-balance the intestinal microecology, so as to affect the occurrence and development of tumors. Moreover, we can also predict the occurrence of tumors by observing the changes in intestinal microorganisms, and improve the effect of chemotherapy and immunotherapy via regulating intestinal microorganisms (Fig. 4).

### 5.1. Dietary regulation

Insufficient ingestion of plant diets, such as vegetables, fruits, and grains, and so on, combined with excessive consumption of meat and greasy foods can lead to an imbalance of dietary structure, which can affect gut microbes and their metabolites, so as to promote the occurrence and development of malignant tumors. A large number of clinical and experimental data have shown that high-fat diet, especially the ingestion of massive saturated fat, which has carcinogenic effect, while obesity caused by high-fat diet is also an important risk factor for a variety of malignant tumors, such as breast cancer, colon cancer, esophageal cancer, thyroid cancer, endometrial cancer, liver cancer, and other cancers.^[83]^ In addition, the carcinogenic mechanisms associated with high-fat diets and gut microbes are mainly achieved by 2 pathways: promoting the increase of intestinal permeability and the introduction of LPS into blood; stimulating the secretion of bile acids and is decomposed into secondary bile acids via intestinal microorganisms.^[84]^ More and more evidence have shown that eating red meat and processed meat is an important risk factor for lung cancer, breast cancer, colorectal cancer, gastric cancer, esophageal cancer, pancreatic cancer, prostate cancer, bladder cancer cancer, and other malignant tumors. In addition to carcinogens such as heterocyclic amines and polycyclic aromatic hydrocarbons produced during high-temperature cooking of meat, choline rich in red meat is a precursor of s-adenosylmethionine, an important methyl donor in the human body. Therefore, differences in the utilization of choline by microorganisms can also affect the epigenetic regulation of the body.^[85]^ For high-protein diets, >95% of proteins are efficiently digested and absorbed in the small intestine, and only a small proportion of proteins that are not completely digested by enzymes enter the large intestine, which can be glycolyzed by the microbiota (such as Bacteroides, Parabacteroides, and so on). Although gut microbes can produce butyric acid that is beneficial to the human body via glycolyzing proteins, they also produce a range of nitrogen compound and sulfur compound, such as ammonia, amines, nitrates, nitrites, hydrogen sulfide, and so on. Among them, amines and nitrites can be catalyzed by anaerobic bacteria in the intestine into N-nitrosamines with strong carcinogenicity, while hydrogen sulfide exists genotoxicity at physiological concentrations. Protein degradation is predominantly located in the distal gut, which is also a highly prevalent site of colorectal cancer.^[86]^

Therefore, changing the structure of unbalanced diet can regulate the intestinal microecology, so as to reduce the production of toxic microbial metabolites, promote the production of protective metabolites, and change the internal environment of the body from cancer-promoting to tumor-inhibiting. For cancer patients, regulating diet is a very effective means of cancer prevention. In a sense, it is also equivalent to treatment without side effects and without increasing the economic burden for cancer patients.

### 5.2. Probiotic ingestion

Probiotics are administered for cancer patients to remodel the gut microbial structure of damaged patients, so as to reestablish the abundance expression and associated function of commensal bacteria that are damaged after treatment. Although probiotics are generally considered safe, there remains some risk of giving probiotics to immunocompromised cancer patients, mainly including the potential risk of acquired infection and transfer of antibiotic resistance.^[87,88]^ Nevertheless, it has been shown in several trials that treatment with probiotics can improve diarrhea and other intestine-related damage caused by antitumor therapy, so as to reconstruct a relatively healthy balance of intestinal microecology.^[89]^ In addition, the Multinational Association of Supportive Care in Cancer and the European Society for Medical Oncology state in clinical practice guidelines for gastrointestinal mucositis that probiotics containing lactobacilli species are recommended to prevent diarrhea after chemotherapy or radiation therapy for pelvic malignancies.^[90,91]^

Notably, early clinical trials are the first time to assess the interaction between probiotic administration, changes in gut microbial composition, and modulation of gut immune function in oncologic patients undergoing colorectal resection. In this double-blind study, patients were treated with a mixture of bifidobacteria and lactobacillus, and it was found that one of LA1 could adhere to the colorectal mucosa, so as to reduce the concentration of intestinal pathogens and regulate local immunity.^[92]^ Subsequently, in 2014, a randomized double-blind controlled trial found that lactobacillus and bifidobacterium could significantly reduce diarrhea caused by pelvic cancer during radiotherapy.^[93]^ In 2015, a clinical trial evaluated the probiotic mixed formula for the first time, Colon Dophilus (a mixture of 10 different probiotic strains) could improve diarrhea caused by irinotecan in patients with metastatic colon cancer during chemotherapy, suggesting that taking such probiotics was relatively safe, and could effectively reduce the incidence and severity of gastrointestinal toxicity caused by diarrhea and chemotherapy.^[94]^ In 2016, another double-blind randomized trial showed that the combination of prebiotics and probiotics for patients undergoing colorectal cancer resection could alleviate irritable bowel syndrome.^[95]^ In 2017, a randomized clinical trial for colon cancer patients showed that perioperative use of a mixture of prebiotics and probiotics could significantly reduce the rate of postoperative infection for colon cancer patients.^[96]^

Although probiotics have achieved appreciable results in gastrointestinal toxicity caused by antitumor treatment, further large-scale controlled clinical trials are needed to truly determine the efficacy and safety of selecting specific species of probiotics during or after antitumor therapy.

### 5.3. FMT

FMT is defined as the transplantation of functional flora in the feces of healthy individuals into the gastrointestinal tract of patients to reconstruct new intestinal flora and achieve the treatment of intestinal and extraintestinal diseases. For example, FMT has been used to treat recurrent Clostridium difficile-infected duodenum.^[97]^ Moreover, FMT has been used in immune rejection-related diseases following allogeneic stem cell transplantation.^[98]^ About the application of antitumor therapy, although pre-clinical studies in mice have demonstrated the effectiveness of FMT in reducing colon tumorigenesis,^[99]^ the effectiveness of its clinical trials still needs to be further confirmed. In addition, several clinical trials aimed at assessing FMT in cancer patients are currently underway, and their common goal is to prevent or ameliorate intestinal toxic side effects associated with antitumor therapy. Although the success of FMT in some trials, there remains a lack of effective management and control in this process, as the entire gut microenvironment is transferred along with therapeutic strains. Notably, the gut microbiota of each person is not exactly the same, but complex and variable, so it is difficult to ensure whether there is immune rejection in the process of the patient’s gastrointestinal tract intrinsic functional flora being transplanted. Therefore, it is very important to strictly control the health status of donors and the special composition of intestinal flora.

### 5.4. Application of Chinese herbal medicines

Chinese herbal medicine is a kind of material from plants, which is mainly treated by oral administration in clinical practice. Due to the advantages of small toxic and side effects in Chinese herbal medicines, it has been remarked by researchers.^[100]^ At present, although research on the active ingredients of Chinese herbal medicines to intervene in tumors is innumerable, research on the treatment of diseases by Chinese herbal medicines through their effects on intestinal microecology remains still in its infancy, and its mechanism is even less clear, which also encourages us from the side to use these Chinese herbal medicines to help treat diseases associated with the intestinal microbiota is very valuable and has a large room for development.

Traditional Chinese medicine (TCM), as a unique ancient medicine, has been widely promoted in Asia for thousands of years by promoting homeostasis to treat various diseases in recent decades. Compared with monotherapy, TCM is characterized by “multi-component” and “multi-target,” so as to have better efficacy for complex diseases. Currently, there is increasing evidence that TCM-specific prescriptions can help patients alleviate the suffering caused by the disease, which suggests that TCM has great potential in clinical treatment. For example, Wenzi Jiedu prescription, a TCM prescription, has been proven to have clinical application value in the treatment of colorectal cancer. As Qiu WL et al^[101]^ found that Wenzi Jiedu prescription had an obvious effect on anticolorectal cancer in vivo and in vitro. Subsequently, the team used network pharmacology to show that it could exert antitumor effect through an approach of “multi-target, multi-pathway.” And according to the analysis of gut microbial flora, this prescription can significantly enrich the tracer to Eubacterium and Bacteroides. Notably, they also found that Wenzi Jiedu prescription significantly increased the proportion of CD8 ^+^ T cells and the expression of immune-related cytokines IL-10, IFN-γ, and TNF-α. These data suggest that the regulation of intestinal flora by compound Chinese traditional medicine (Wenzi Jiedu prescription) may be a breakthrough to elucidate its functional mechanism in the treatment of colorectal cancer, so as to have a good prospect of clinical application. Here, we combed these prescriptions to elucidate that prescriptions composed of multiple Chinese herbal medicines influence the development and progression of certain diseases by modulating the intestinal microecological flora (Table 1).

**Table 1:** Effect of TCM prescription mediated-gut flora in the development and progression of diseases.

Prescription	Formation	Signaling pathway or targets	Involved gut flora	Related diseases	References
Wenzi Jiedu Recipe	*Astragali Preparata, Atractylodes, Coicis Semen, Agrimonia pilosa, Sparganii Rhizoma, Rhizoma Curcumae, Sophorae Flavescentis Radix, Coptidis Rhizoma*	IL-10, IFN-γ, TNF-α	*Oscillibacter*, *Bacteroides*	Colorectal cancer	^[101]^
Dingxin Recipe IV	*Coptis chinensis Franch, Salvia miltiorrhiza Bunge, Ziziphus jujuba Mill, Ganoderma lucidum*	LXR-α/SREBP1	*Firmicutes*, *Bacteroidetes, Alloprevotella, Allobaculum, Ileibacterium*	Atherosclerosis	^[102]^
Huangqin decoction	*Scutellaria, Peony*, *Liquorice, Jujube*	PI3K/AKT, TLR4	*Lactobacillus*, *Firmicutes*, *Bacteroides*, *Treponema*, *Bacteroides*	Ulcerative colitis	^[103]^
Sanziguben	*Rosae laevigatae Micx, Phyllanthus emblica L, Schisandra chinensis Baill, Gynostemma pentaphyllum Makino*	TLR4/NF-κB/NLRP3	*Proteobacteria, Klebsiella, Escherichia-Shigella*	Diabetic nephropathy	^[104]^
FuFang Zhenshu TiaoZhi	*Citri sarcodactylis fructus, Ligustri lucidi fructus, Salviae miltiorrhizae radix et rhizoma, Notoginseng radix et rhizoma, Coptidis rhizoma, Atractylodis macrocephalae rhizoma, Cirsii japonici herba et radix, Eucommiae cortex*	TNF-α, IL-6, IL-8	*Firmicutes*, *Bacteroidetes, Lactobacillus murinus, Erysipelatoclostridium, Butyricimonas, Clostridium, Akkermansia*	Aging	^[105]^
Byur dMar Nyer lNga Ril Bu	*Terminalia chebula Retz.*, Carthamus tinctorius *L.*, Glycyrrhiza glabra *L., Swertia bimaculata, Agilawood, Acorus calamus L.*	Aß, p-tau	*Muribaculum, Bacteroidales, Ruminococcus-1, Ruminiclostridium-9*	Alzheimer disease	^[106]^
Baitouweng decoction	*Radix pulsatilla, Cortex phellodendri, Rhizoma coptidis, Cortex fraxini*	IL-6/STAT3	*Firmicutes*, *Bacteroidetes, Proteobacteria, Escherichia-Shigella, Lactobacillus, Akkermansia*	Ulcerative colitis	^[107]^

TCM = traditional Chinese medicine.

Notably, in addition to the study of prescriptions, the related studies of active ingredients in single herbs are also favored by researchers. Many compounds can be extracted from Chinese herbs and used to treat related diseases in vivo or in vitro and in clinical trials. For example, *Coptis chinensis Franch* is the dry rhizome of Ranunculaceae plants, which is rich in berberin, tetrandrine, and other active ingredients.^[108]^ Sun Q et al^[109]^ found that Berberine could inhibit the growth of colorectal tumor cells, induce apoptosis, and arrest cell cycle by decreasing the expression of Hedgehog signaling pathway, so as to improve the pathological manifestations in mouse with colorectal cancer mouse. In addition, berberine can also significantly reduce β-diversity of gut microbiota in mice with colorectal tumors, while without affecting α-diversity. Meanwhile, probiotic microorganisms were enriched by gut microbiota analysis, while pathogenic microorganisms showed a decreasing trend, indicating that berberine can also modulate the function of gut microbiota in mice with colorectal tumors. *Ginseng* is the dried rhizome of Araliaceae plant and contains active components, such as ginseng polysaccharides, ginsenosides, panaxenol, and so on.^[110]^ Huang JM et al^[111]^ found that the Ginseng polysaccharide combined with α-PD-1 monoclonal antibody may be a novel strategy that increases the sensitivity of patients with non-small cell lung cancer to anti-α-PD-1 immunotherapy. In addition, this team combined gut microbiota analysis to show that the abundance expression of Bacteroides species in the combined treatment group was much higher than the α-PD-1 alone and control groups, suggesting that the gut microbiota can be used as a new biomarker to predict the response of anti-PD-1 immunotherapy. Here, we summarized these compounds to elucidate the effect of active ingredients from Chinese herbs on the development and progression of certain diseases by regulating the intestinal microecological flora (Table 2).

**Table 2:** Compounds from Chinese herbs intervened the development and progression of diseases by regulating the gut flora.

Compound	Chinese medicine	Signaling pathway or targets	Involved gut flora	Related diseases	References
Berberine	Coptis chinensis Franch	Hedgehog	Probiotic microbes, pathogenic microbes	Colorectal cancer	^[109]^
Ginseng polysaccharides	Ginseng	PD1/PDL-1	Parabacteroides distasonis, Bacteroides vulgatus	Non small lung cancer	^[111]^
Total flavonoids	Glycyrrhiza uralensis	TNF-α, IL-6β, IL-11, NLRP3	Lactobacillus	Colitis	^[112]^
Salvia-Nelumbinis naturalis	Salvia	FXR/FGF15	Firmicutes, Bacteroidetes	Nonalcoholic steatohepatitis	^[113]^
Allicin	Garlic	Cd14/TLR4, TNF-α, IL-4β, IL-14	Firmicutes, Bacteroidetes	Alcoholic steatohepatitis	^[114]^
Diosmetin	Dioscorea oppositifolia L.	IRS/PI3K/AKT	Corynebacterium glutamicum	Type 2 diabetic mellitus	^[115]^
Curcumin	Curcuma longa L.	IL-6/Stat3/c-Myc	Akkermansia, Bacteroides	Colitis-associated tumorigenesis	^[116]^
Astragalus polysaccharide	Astragalus	TLR4/ NF-κB	Probiotics	Idiopathic pulmonary fibrosis	^[117]^
Resveratrol	Polygonum cuspidatum	NF-κB, IL-6, TNF-α, MCP-1	Firmicutes, Actinobacteria, Proteobacteria, Bacteroidetes	Acute pancreatitis	^[118]^
Salidroside	Rhodiola rosea L.	IL-1β, NLRP3, Caspase-1, GSDMD p30	Firmicutes	Ulcerative colitis	^[119]^
Lycium barbarum polysaccharide	Lycium barbarum Leaves	TNF, IL-4, IL-6, MCP-1, IL-17A	Lactobacillus, Bifidobacterium, Firmicutes, Actinobacteria, Alistipes, Clostridiales	Asthma	^[120]^
Neohesperidin	Citrus reticulata Blanco.	Wnt/β-catenin	Bacteroidetes, Firmicutes, Proteobacteria	Colorectal cancer	^[121]^
Paeonol	Tree Peony Bark	SOD, MDA	Lactobacillus	Gastric ulcers	^[122]^
Diosgenin	Dioscorea oppositifolia L.	PD-1, IFN-γ, PARP, Caspase-3	Lactobacillus, Sutterella, Bacteroides	Melanoma	^[123]^
Angelica sinensis polysaccharide	Angelica sinensis	Cldn5, Slit3, Rgs18	Clostridia, Lactobacillus, Oscillospiraceae, Desulfovibrionacea	Rheumatoid arthritis	^[124]^

### 5.5. Other strategies

More and more evidences have shown that the occurrence and development of tumors are closely related to the changes in intestinal microecology. As suggested by Zitvogel L et al,^[125]^ 9 major cancers (including acute lymphoblastic leukemia, head and neck squamous cell carcinoma, oral cancer, lung cancer, breast cancer, pancreatic cancer, cholangiocarcinoma, cervical cancer, and urothelial carcinoma) are associated with specific members of gut microecological members and levels of abundance expression. In addition, Dai et al^[126]^ have found that 7 bacteria (such as Bacteroides fragilis, Fusobacterium nucleatum, and so on) are enriched in the intestines of colorectal cancer patients by metagenomic analysis of 526 stool samples from 5 different countries, and another 62 bacteria are in a decreasing trend, and these potential diagnostic bacterial markers can be used for noninvasive diagnosis of colorectal cancer. Other studies have shown that microbial characteristics related to early lung cancer have been found in the systematic changes of intestinal flora in patients with lung cancer. In order to construct clinical indicators for early diagnosis by using sequencing or real-time quantitative PCR, the research team develops a reference-based biomarker development strategy, an OTU-based high-precision predictor for early diagnosis of lung cancer, and this method for early lung cancer diagnosis will bring more hope to the majority of lung cancer patients if it passes clinical trials.^[55]^

When microorganisms are affected by dysbacteriosis, they can have a profound impact on the pathogenesis of tumors and their therapeutic outcome. In particular, the adjustment of treatment outcomes is closely related to gut microbial metabolism, antitumor drugs, and the ability to modulate host’s immune response and inflammatory pathways. The combination of these 2 effects can explain why the intestinal microbial composition of patients can significantly affect the efficacy of chemotherapy and immunotherapy. In aspect of chemotherapy, it is reported that tumor-bearing mice, either sterile or tumor-bearing mice with reduced intestinal microbiota after antibiotic treatment, have no response to oxaliplatin treatment. The reason for this may be that commensal microbiota in the mouse gut produce TLR agonists, which promote the elevation of oxidative stress environment and the death of tumor cells. However, without a healthy gut microbiota, microbiota-dependent ROS production is reduced and the response to chemotherapy also becomes weaker.^[127]^ Another study has found that mice with lung cancer treated with a combination of cisplatin and antibiotics have a lower survival rate and are more likely to form larger tumors. If cisplatin is combined with probiotics (such as Lactobacilli), it can increase the response of tumor-bearing mice to treatment, and the mechanism may be associated with the induction of pro-apoptotic genes within the tumor and the enhancement of the host immune response.^[128]^ In addition, another widely used chemotherapeutic drug, cyclophosphamide, in combination with probiotic mixtures (Lactobacillus illusjohonsoni and Enterococcus hirae), can promote the conversion of T cells from an immature state to pro-inflammatory Th17 cells, and ultimately improve the therapeutic effect of cyclophosphamide in tumor-bearing mice.^[129]^

In aspect of immunotherapy, administration of CpG oligonucleotides (a synthetic small molecule that mimics bacterial DNA) can significantly stimulate the body’s immune system, so as to show good activity of antitumor in several malignant tumors.^[130]^ It has been found that intratumoral injection of CpG oligonucleotides combined with IL-10R antibody can induce TNF production by myeloid cells associated with tumor infiltration, so as to inhibit the growth of many types of tumors in mice. Moreover, when antibiotic-treated tumor-bearing mice were injected with a particular bacterium (such as Alistipes shahii), the TNF production could be restored and the therapeutic outcomes could be significantly improved.^[127]^

## 6. Vision of the future

More and more evidence shows that there is a relationship between intestinal microecology and incidence rate of tumors. Studies show that intestinal microecology interacts with the host in various ways. Abnormal gut microbiota composition or microbial metabolites may be responsible for altering cancer risk and its associated pathological alterations. Therefore, the potential use of intestinal microecology can not only predict the occurrence of early tumors and improve the efficacy of chemotherapy or immunotherapy but also develop new strategies for cancer prevention and treatment.

Although many in vitro cell experiments or in vivo animal experiments have achieved success in treating some malignant tumors by regulating the homeostasis of intestinal microecology, their clinical trials are currently intensifying, and the related mechanisms have not been elucidated. In order to promote the potential application of intestinal microecology in malignant tumors, the following issues urgently need to be solved in the future: First of all, it is necessary to identify specific microbial strains, rather than general bacterial communities, which will help elucidate the contribution of specific microbes to tumor progression. Secondly, based on the current situation where research mainly explores microbial composition, future research directions may be more focused on the functions of metabolites modified by microorganisms and their downstream factors. Thirdly, due to the microbiota in each patient having complex and variable properties, we urgently need personalized microbiota modification methods, an effort that may help individual patients to accurately determine and analyze the microbiota of metabonomics biomarkers. Fourthly, in order to further investigate the complex mechanism between intestinal microecology and host, we can use advanced technologies (such as nanomedical technology, data science, and the inclusion of race, gender, and other factors) to clarify the mechanism mediated by intestinal bacteria, which can guide us to formulate more effective and high-precision tumor prevention and treatment programs, and ultimately reduce the social and economic burden of tumor patients.

## Acknowledgments

The authors sincerely thank the funding of the Open Foundation of Northwest Collaborative Innovation Center for Traditional Chinese Medicine Co-constructed by Gansu Province &MOE of PRC (No. xbzzy-2022-05) and the Lanzhou Science and Technology Plan Project (No. 2022-3-40).

## Author contributions

**Conceptualization:** Yan Zhang.

**Formal analysis:** Le-Le Tian, Ying Zhou, Jing-Quan Teng.

**Funding acquisition:** Xu-Dong Lei, Yan Zhang.

**Writing – original draft:** Jin-Ping Qian, Bing Jiang, Xu-Dong Lei.

**Writing – review & editing:** Jia Yue, Jin-Juan Li.
